# How Many Is Enough? Effect of Sample Size in Inter-Subject Correlation Analysis of fMRI

**DOI:** 10.1155/2016/2094601

**Published:** 2016-01-13

**Authors:** Juha Pajula, Jussi Tohka

**Affiliations:** ^1^Department of Signal Processing, Tampere University of Technology, P.O. Box 553, 33101 Tampere, Finland; ^2^Department of Bioengineering and Aerospace Engineering, Universidad Carlos III de Madrid, Avenida de la Universidad 30, 28911 Leganes, Spain; ^3^Instituto de Investigacion Sanitaria Gregorio Marãnon, Calle de Doctor Esquerdo 46, 28007 Madrid, Spain

## Abstract

Inter-subject correlation (ISC) is a widely used method for analyzing functional magnetic resonance imaging (fMRI) data acquired during naturalistic stimuli. A challenge in ISC analysis is to define the required sample size in the way that the results are reliable. We studied the effect of the sample size on the reliability of ISC analysis and additionally addressed the following question: How many subjects are needed for the ISC statistics to converge to the ISC statistics obtained using a large sample? The study was realized using a large block design data set of 130 subjects. We performed a split-half resampling based analysis repeatedly sampling two nonoverlapping subsets of 10–65 subjects and comparing the ISC maps between the independent subject sets. Our findings suggested that with 20 subjects, on average, the ISC statistics had converged close to a large sample ISC statistic with 130 subjects. However, the split-half reliability of unthresholded and thresholded ISC maps improved notably when the number of subjects was increased from 20 to 30 or more.

## 1. Introduction

Inter-subject correlation (ISC) [1, 2] is a widely used method for detecting and comparing activations in functional magnetic resonance imaging (fMRI) acquired during complex, multidimensional stimuli such as audio narratives, music, or movies [3–9]. Instead of trying to model the stimulus as in the standard general linear model (GLM) based fMRI analysis ISC computes voxel-by-voxel correlations of the subjects' fMRI time courses, assuming that the images have been registered to a common stereotactic space. The activation maps can then be formed by thresholding the average correlation coefficient values. The ISC method has been shown to produce activation maps closely matching those of the standard GLM based analysis when the stimuli are simple and can be modelled [10]. Note, however, that while not using a model time course of the stimulus, ISC expects that all the subjects are exposed to the same stimulus and it is not a method for an analysis of resting state fMRI.

A common challenge in any fMRI group analysis, including ISC analysis, is to define the required number of subjects in such a way that the analysis results are reliable and have enough statistical power, but the costs of the data acquisition are minimized. In principle, a larger sample size provides a more reliable analysis and more statistical power [11, 12]. Obviously, the sample size is not the only factor contributing to reliability (or the statistical power) of the study, but ideally the whole study design should be done to reach the desired limits of statistical power [13–15]. However, between-subject variability in fMRI data is generally much higher than within-subject variability and consequently choosing a large enough sample size is essential [16].

While there are no general methods for the optimal experimental design using naturalistic stimuli, the generalizability of the analysis results, necessarily with a limited sample size, to the population level is an important consideration. Particularly, it is important to know how many subjects are required for a reproducible (or reliable) analysis, so that small variations in the subject sample do not cause too large variations in the analysis results. This is the question we ask in this paper and to our knowledge it has not been addressed previously in the context of the ISC analysis. Similar studies on the reliability of fMRI group studies with general linear model (GLM) analyses have been reported earlier in [16–18]. All of these studies have concluded that closer to 30 subjects should be included in a group level studies in fMRI data analysis. The sample size issue has been studied also with independent component analysis [19], where the reproducibility of the results was noticed to improve with an increased number of subjects. Critically, David et al. [20] reported that the average number of subjects in their meta-analysis was 13 and 94% of all studies were applied with less than 30 subjects, which suggests that typically fMRI group studies based on GLM might not reach the required level of reliability.

In this study, we examined how the number of subjects included in the study affects the reliability of the statistical ISC maps and the FDR corrected binary thresholded maps. We used a large 130-subject data set with a simple block design task and performed a split-half resampling based analysis (similar to [16]) while varying the number of subjects in each split-half. The resampling procedure was repeated 1000 times. This setup enables us to address the reproducibility of the studies with the maximum of 65 subjects. We compared the statistical ISC maps formed using independent subjects samples and also the thresholded ISC maps. In addition and similarly to [17] we compared statistical ISC maps with the subsets of 130 subjects with the statistical ISC map derived from the whole 130-subject data set.

## 2. Materials and Methods

### 2.1. fMRI Data

The fMRI data used in the preparation of this work were obtained from the ICBM database (https://ida.loni.usc.edu/login.jsp?project=ICBM) in the Image Data Archive of the Laboratory of Neuro Imaging. The ICBM project (Principal Investigator John Mazziotta, M.D., University of California, Los Angeles) is supported by the National Institute of Biomedical Imaging and BioEngineering. ICBM is the result of efforts of coinvestigators from UCLA, Montreal Neurologic Institute, University of Texas at San Antonio, and the Institute of Medicine, Juelich/Heinrich Heine University, Germany.

We selected all subjects from the ICBM database who had fMRI measurements with the verb generation (VG) task and the structural MR image available. This produced 132 subjects' data set. After a quality check by visual inspection two subjects were discarded due to clear artifacts in their fMRI data. This led to a final data set of 130 subjects: 61 males, 69 females; age range 19–80 years, mean 44.35 years; 117 were right-handed, 10 were left-handed, and 3 were ambidextrous. The data was acquired during the block design VG task (a language task with a visual input) from Functional Reference Battery (FRB) developed by the International Consortium for Human Brain Mapping (ICBM) [21]. The FRB holds a set of behavioral tasks designed to reliably produce functional landmarks across subjects and we have previously used fMRI data extracted from the ICBM FRB database for other experiments [10, 22]. The details of the data and VG task are provided in [10]. The VG task contained the largest number of subjects with fMRI measurements in the ICBM database among the five FRB tasks and therefore we selected it for this study.

The functional data was collected with a 3-Tesla Siemens Allegra fMRI scanner and the anatomical T_1_ weighted MRI data was collected with a 1.5-Tesla Siemens Sonata scanner. The TR/TE times for the functional data were 4 s/32 ms, with flip angle 90 degrees, pixel spacing 2 mm, and slice thickness 2 mm. The parameters for the anatomical T_1_ data were 1.1 s/4.38 ms, 15 degrees, 1 mm, and 1 mm, correspondingly.

### 2.2. Preprocessing

The preprocessing of the data was performed with FSL (version 5.0.2.2) from Oxford Centre for Functional Magnetic Resonance Imaging of the Brain, Oxford University, Oxford, UK [23]. The data preprocessing, which was identical to [10], included motion correction with FSL's MCFLIRT and the brain extraction for the functional data was done with FSL's BET [24]. The fMRI images were temporally high-pass filtered with a cutoff period of 60 s and the spatial smoothing was applied with an isotropic three-dimensional Gaussian kernel with the full-width half-maximum (FWHM) 5 mm in each direction. The brain extraction of the structural T_1_ images was also performed by BET, but this was done separately from the main procedure for each T_1_ weighted images as the parameters of BET required individual tuning for the images.

The image registration was performed with FSL Linear Registration Tool (FLIRT) [25, 26] in two stages. At the beginning, the skull-stripped functional images were aligned (6 degrees of freedom, full search) to the skull-stripped high-resolution T_1_ weighted image of the same subject, and then the results were aligned to the standard (brain only) 2 mm ICBM-152 template (12 degrees of freedom, full search).

### 2.3. ISC Analyses

All of the ISC analyses were computed with ISCtoolbox for Matlab [2]. ISCtoolbox computes the ISC statistic by first computing Pearson's correlations between the corresponding time series of all subject-pairs. Then, to obtain the final multisubject test statistic, correlation values of all subject-pairs are combined into a single ISC statistic by averaging. This is the ISC statistical map.

The statistical inference was accomplished by a fully nonparametric voxel-wise resampling test implemented in the ISCtoolbox [27]. The resampling test constructs the null-distribution of the ISC values by circularly shifting the time series of each subject by a random amount. This test resembles the circular block bootstrap test [28] and it accounts for temporal correlations inherent to fMRI data. For a more detailed description of the test, we refer to [29]. For thresholding each ISC map, the resampling distribution was approximated with 10 000 000 realizations, sampling randomly across the brain voxels for each realization and generating a new set of time-shifts (one for each subject) for each realization. The resulting *p*-values were corrected voxel-wise over the whole brain using a false discovery rate (FDR) based multiple comparisons correction [30].

### 2.4. Experimental Procedure

We performed a split-half resampling type of the analysis for the ISC method. The process consisted of randomly drawing (without replacement) two independent subsets of *P* = 10,15,…, 65 subjects from the total pool of 130 subjects. Then, the full ISC analysis (including resampling distribution approximation and computation of corrected thresholds) was performed for both subsets and the full ISC analysis results from both sets were saved. This process was repeated 1000 times meaning that the ISC analysis was performed separately and independently 2000 times for each number of subjects *P* = 10,15,…, 65.

We compared the ISC statistical maps of the split-half analysis with the following criteria.

(1) Pearson's correlation coefficient *C*_*n*_ for comparing the nonthresholded statistical maps was defined as

(1) $$\begin{array}{c} C_{n}=\frac{1}{K-1}\overset{K}{\underset{k=1}{\sum }}\left(\;\frac{{\overset{-}{l}}_{k}-\overset{-}{L}}{s_{\overset{-}{l}}}\;\right)\left(\;\frac{{\overset{-}{r}}_{k}-\overset{-}{R}}{s_{\overset{-}{r}}}\;\right), \end{array}$$

where *K* is the total number of brain voxels in the volume. ${\overset{-}{l}}_{k}$ and ${\overset{-}{r}}_{k}$ are the two ISC statistics of the *k*th voxel, respectively. $\overset{-}{L}$ and $\overset{-}{R}$ are the sample means of $\left\{{\overset{-}{l}}_{k}\right\}$ and $\left\{{\overset{-}{r}}_{k}\right\}$ across the brain volume, and $s_{\overset{-}{l}}$ and $s_{\overset{-}{r}}$ are the standard deviations of $\left\{{\overset{-}{l}}_{k}\right\}$ and $\left\{{\overset{-}{r}}_{k}\right\}$ across the brain volume. The final measure was computed by averaging the correlation measures *C*_*n*_ according to

(2) $$\begin{array}{c} C_{avg}=\frac{1}{N}\overset{N}{\underset{n=1}{\sum }}C_{n}, \end{array}$$

where *N* is the number of resampling replications, which was 1000 in this study.

(2) The mean absolute error (MAE) between paired ISC maps was defined according to

(3) $$\begin{array}{c} M_{n}=\frac{1}{K}\overset{K}{\underset{k=1}{\sum }}\left|\;{\overset{-}{r}}_{k}-{\overset{-}{l}}_{k}\;\right|, \end{array}$$

where *K* is the total number of brain voxels in the volume. ${\overset{-}{r}}_{k}$ and ${\overset{-}{l}}_{k}$ are the two ISC statistics of the *k*th voxel, respectively. The final measure was computed by averaging the MAE measures *M*_*n*_ according to

(4) $$\begin{array}{c} M_{avg}=\frac{1}{N}\overset{N}{\underset{n=1}{\sum }}M_{n}, \end{array}$$

where *N* = 1000 is the number of resampling replications.

We used Dice index to compare the thresholded paired binary ISC activation maps [31]. The justification for the use of Dice index can be found in [10]. The Dice index between two sets (*A*_*n*_ and *B*_*n*_, *n* = 1,…, 1000 refers to resampling replication) of activated voxels was defined as

(5) $$\begin{array}{c} D_{n}=\frac{2\left|A_{n}\cap B_{n}\right|}{\left|A_{n}\right|+\left|B_{n}\right|} \end{array}$$

and it takes values between 0 and 1. The tested thresholds were corrected with a false discovery rate (FDR) over the whole brain using *q* = 0.05, *q* = 0.01, and *q* = 0.001 (no correlation assumptions). The Dice indexes were computed for 1000 times for each number of subjects and the reported average Dice index was computed by averaging 1000 Dice indexes *D*_*n*_ in the same way as with correlation and MAE measures.

The Dice index defines the binary similarity between two binary images and it can be categorized with Landis and Koch categorization for Kappa coefficients [10]. According to [32] the categories are

- ≤0, no agreement,
- 0–0.2, slight agreement,
- 0.2–0.4, fair agreement,
- 0.4–0.6, moderate agreement,
- 0.6–0.8, substantial agreement,
- 0.8–1.0, almost perfect agreement.

As Landis and Koch themselves note these categories are highly subjective [32] but are maybe useful as a reference.

Similarly to [17], we considered how fast the statistic maps converge to a large sample statistic map with 130 subjects. For this, we repeated Pearson's correlation analyses described above by comparing statistic maps resulting from resampling to the statistic map obtained using all 130 subjects as in (1) and averaging over 2000 resampling iterations. More specifically, $\overset{-}{r}$ and $\overset{-}{R}$ in (1) were from the same statistic map with 130 subjects and in (2) *N* was then 2000. We computed also the sensitivity and specificity of thresholded ISC maps by using the thresholded 130 subjects ISC statistic with the corresponding threshold (*q* = 0.05, *q* = 0.01, and *q* = 0.001 with no correlation assumptions) as the ground truth. The final sensitivity and specificity (for each number of subjects) were averaged from 2000 sensitivity and specificity measures that resulted from 1000 split-half resampling replications.

### 2.5. Implementation

This study was computationally demanding. For each number of subjects, 2000 ISC analyses with 10 000 000 realizations for corrected thresholds were computed. This was repeated with 12 different numbers of subjects and the whole analysis required 24 001 ISC analyses (one extra analysis was for the whole data set of 130 subjects). For implementing the computations, parallel computing environment Merope of Tampere University of Technology, Finland, was used. It has nodes running on HP ProLiant SL390s G7 equipped with Intel Xeon X5650 CPU 2,67 GHz and minimum of 4 GB RAM/core. The used grid engine was Slurm. The equivalent computing time would have been 4.75 years if they had been computed with a single high end CPU.

## 3. Results

**Figure 1:**
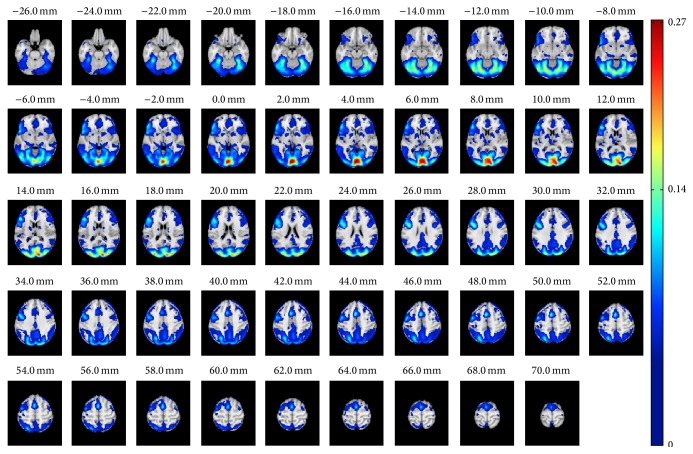
The ISC analysis based on 130 subjects. The figure presents the axial slices of the ISC analysis results of the whole 130 subjects' data set after applying FDR corrected *q* = 0.001 thresholding. The full statistical map is visible and available in NeuroVault: http://www.neurovault.org/collections/WTMVBEZP/images/11576/.

Figure 1 presents the thresholded (voxel-wise FDR corrected over the whole brain *q* = 0.001) results from the ISC analysis with the whole 130 subjects' data set. Significant ISC values were found around occipital and temporal lobes, lateral occipital cortex, and paracingulate gyrus as well as on middle frontal and inferior frontal gyri. The 130-subject ISC map was highly similar to ISC map presented earlier with partially the same data but with smaller number of subjects (*P* = 37) [10]. The most noticeable difference compared with the 37-subject analysis was that with 130 subjects a larger number of voxels survived from the threshold and significant ISCs formed a more symmetric pattern over the hemispheres. One specific note concerning ISC map of Figure 1 is in order: There appears to be an artifact, which can be seen as a thin activation line in the left frontal cortex (e.g.) in the axial slice *z* = 50 mm. The investigation of the data at that location revealed a slight signal drop in time series of majority of subjects, buried under the noise in any single subject data, which increased ISC values with the large data set to level of statistical significance. The temporal location of the drop was in the middle of the time series (*t* = 172 s, while not counting the stabilization volumes). The statistical ISC map from 130 subjects is available in the NeuroVault service [33] at http://www.neurovault.org/collections/WTMVBEZP/images/11576/.

**Figure 2:**
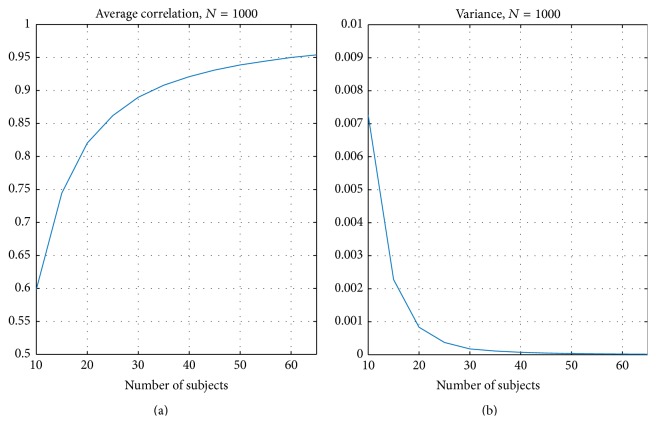
Average correlation *C*_avg_ over 1000 resampling replications. (a) presents the average correlation over and (b) the corresponding variance of *C*_*n*_,  *n* = 1,…, 1000. The correlation increased when the sample size increased and at the same time the variance decreased.

Figure 2 presents the correlation criteria resulting from the split-half resampling analysis.  Figure 2(a) presents the average correlation *C*_avg_ (2) and Figure 2(b) presents the corresponding variance of *C*_*n*_,  *n* = 1,…, 1000 (see (1)). As expected the average correlation between nonoverlapping samples increased when the number of subjects increased and, at the same time, the variance decreased. The average correlation curve was not linear with respect to the number of subjects and stabilized after 30 subjects finally reached the value of 0.95 as the number of the subjects reached the value of 65.

**Figure 3:**
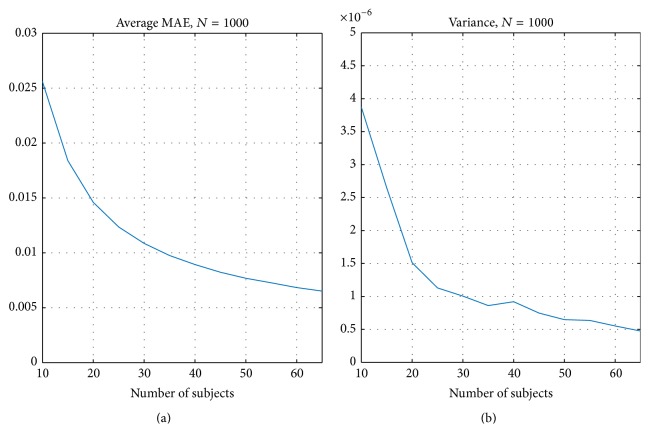
Average MAE *M*_avg_ over 1000 resampling replications. (a) presents the average MAE and (b) the corresponding variance of *M*_*n*_,  *n* = 1,…, 1000.

**Figure 4:**
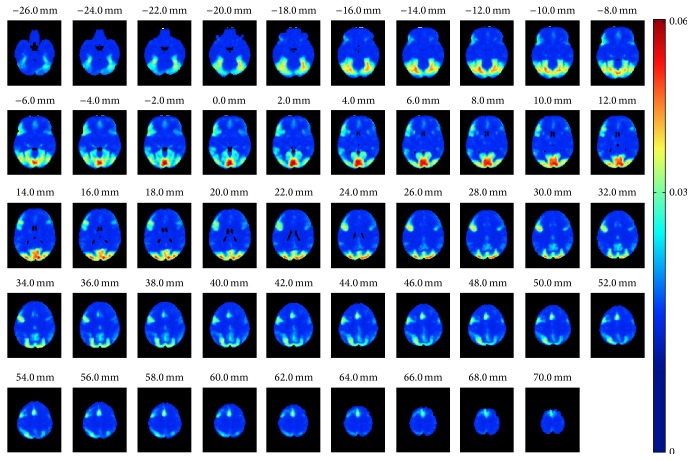
Average MAE computed voxel-wise over 1000 resampling replications with 30 subjects. The average voxel-wise MAE map had similar spatial shape with every tested number of subjects. The only clear difference was the magnitude of MAE values.

Figure 3 presents the MAE criteria resulting from the split-half resampling analysis.  Figure 3(a) presents the average MAE (3) and Figure 3(b) presents the corresponding variance of *M*_*n*_,  *n* = 1,…, 1000 (see (3)). Again, as expected, the average MAE between nonoverlapping samples decreased when the number of subjects increased and at the same time the variance decreased, largely replicating the correlation based curves in Figure 2. With 20 subjects the average MAE was 0.015 and with 30 subjects it was 0.011 indicating that, on average, ISC with 20 or 30 subjects already provided a high degree of reproducibility when averaged over the whole brain. However, this does not reveal whether there were variations in the reproducibility in voxel-wise ISC values across the brain.  Figure 4 presents how the MAEs were distributed over the brain volume with 30 subjects. We note that the spatial shape of MAE distribution across the brain was highly similar to all numbers of subjects, and only the magnitude of the average MAE changed. Comparing Figure 4 with Figure 1 revealed that the highest variations in the ISCs coincided with the highest ISC values. The three-dimensional MAE maps with all numbers of subjects are available in the NeuroVault service [33] at http://www.neurovault.org/collections/WTMVBEZP/.

**Figure 5:**
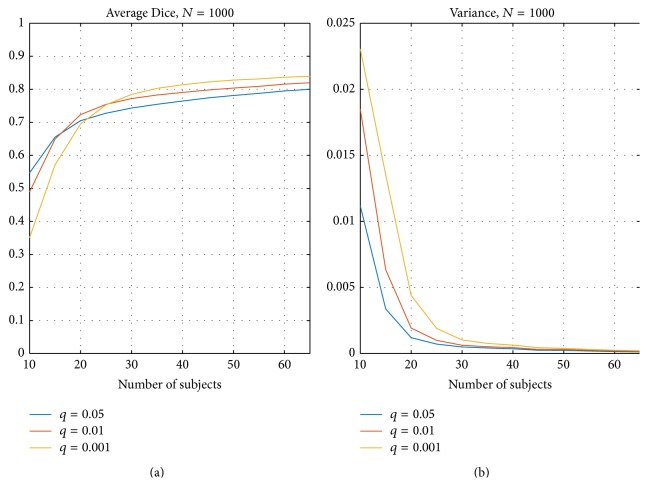
Average Dice index over 1000 resampling replications with three FDR levels: *q* = 0.05, *q* = 0.01, and *q* = 0.001. (a) presents the average Dice indexes *D*_*n*_ over 1000 replications and (b) presents the corresponding variance. The curve corresponding to the most conservative threshold *q* = 0.001 (yellow) shows that more subjects are required for greater similarity after applying the threshold to the data. The more liberal thresholds *q* = 0.01 (in red) and *q* = 0.05 (in blue) required fewer subjects to stabilize than the most conservative threshold *q* = 0.001 (yellow) but on the other hand the highest similarity was reached with the most conservative threshold.

Figure 5 presents Dice indexes over the 1000 resampling replications.  Figure 5(a) presents the average of Dice indexes *D*_*n*_ for three threshold levels (voxel-wise FDR corrected over the whole brain with *q* = 0.05 (blue), *q* = 0.01 (red), and *q* = 0.001 (yellow)).  Figure 5(b) presents the corresponding variance of the Dice indexes *D*_*n*_. Again, as expected the Dice similarity between thresholded ISC maps increased when the number of subjects increased and the variance of Dice indexes decreased when the number of subjects increased. Based on Figure 5(a), it is noticeable that more conservative thresholds required slightly more subjects to stabilize. The most liberal threshold *q* = 0.05 had all average Dice indexes within the category “substantial agreement” but stays under the level of “almost perfect agreement” even with 65 subjects. The more conservative *q* = 0.01 reached the “almost perfect agreement” level with 45 subjects and *q* = 0.001 had the Dice index over the required 0.8 already with 35 subjects.

**Figure 6:**
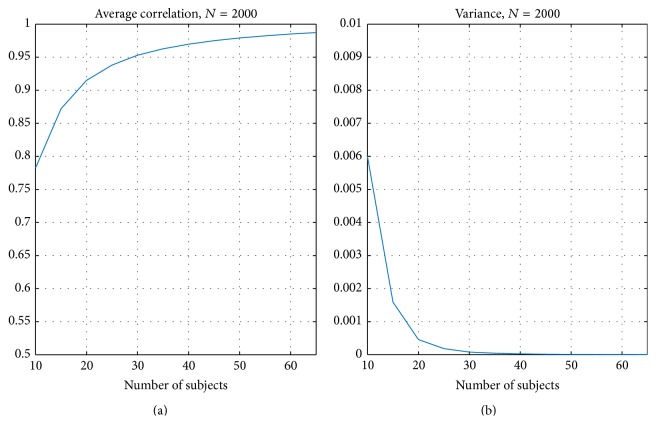
Average correlation comparing subsampled ISC maps with the ISC statistic map of the whole 130 subjects. (a) presents the average correlation over 2000 replications and (b) presents the corresponding variance. Again, the correlation increased when the number of subjects increased. With 30 subjects or more, the average correlation was greater than 0.95 and the variance was less than 0.0002.

**Figure 7:**
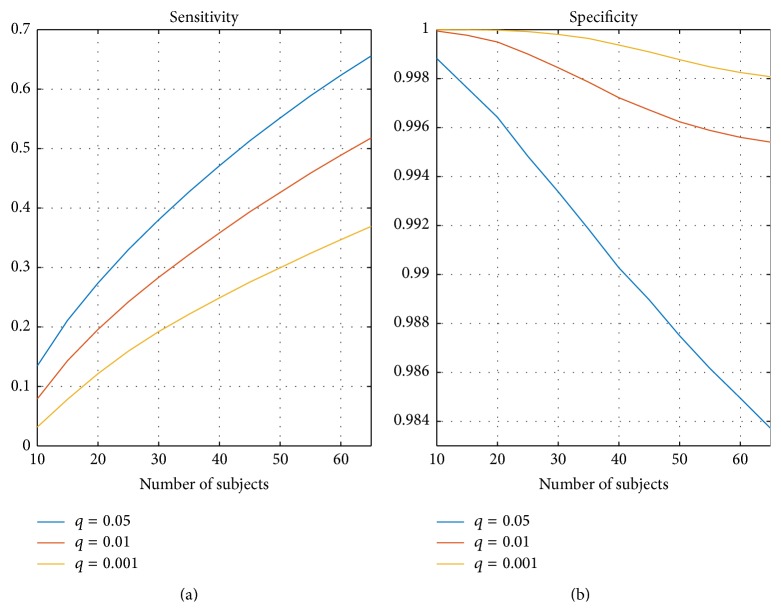
Average sensitivity and specificity from thresholded binary maps compared with the thresholded ISC statistic map of the full 130-subject sample size. (a) presents the average sensitivity over *N* = 2000 replications and (b) presents the corresponding specificity. The sensitivity increased when the number of subjects increased. The specificity was close to 1 with conservative thresholds and even with the most liberal threshold with *q* = 0.05 the specificity was over 0.98 with any number of subjects.

Figure 6 presents the average of correlation when ISC maps with resampled subsets of subjects were compared with the ISC map computed with the whole set of 130 subjects (average over 2000 resampling replications). In Figure 6, (a) presents the average correlation and (b) presents the corresponding variance. Again, the correlation increased when the number of subjects increased and the variance decreased when the number of subjects increased. The variance was close to zero and the correlation to the full 130-subject ISC map was 0.95 with 30 subjects. The sensitivity and specificity curves, using 130-subject thresholded ISC map as the ground truth, are presented in Figure 7. The sensitivity increased when the number of subjects increased and the specificity stayed close to 1 with all numbers of subjects. Figure 7 also shows that the more liberal the threshold the higher the sensitivity value at a slight expense of the specificity value.

## 4. Discussion

In this study, we evaluated the reliability of the ISC analysis for fMRI data and studied the effect of the sample size on the reliability of the ISC analysis. This was accomplished by using a split-half resampling based design, similar to that of [16]. We randomly sampled two nonoverlapping subsets of subjects from the 130-subject ICBM-fMRI data set with a verb generation task. We iterated the paired resampling procedure 1000 times for each number of subjects varying from 10 to 65 and compared the ISC analysis results obtained based on two nonoverlapping subsets of subjects. We compared both the raw ISC statistic maps and the thresholded statistical maps.

Previously, we have validated the ISC analysis against a gold standard set by GLM analysis in [10] and investigated the effect of smoothing to the ISC analysis results in [22]. Both of these studies used a relatively large fMRI data set of 37 subjects, which was larger than the data sets typically applied in the naturalistic stimulus experiments. Therefore, in addition to the question concerning the reliability of the ISC analysis, it was important to study how many subjects are needed for the ISC analysis in order for statistical maps to stabilize. When comparing the ISC results of our earlier study applied for 37 subjects [10] with the current study of 130 subjects, it is not surprising that the statistical power of the analysis had been increased with the increased number of subjects; that is, the activated areas were larger with 130 subjects.

When examining the voxel-wise MAE values shown in Figure 4, it was clear that the largest MAE coincided with the strongest ISCs in Figure 1. This is an interesting phenomenon because purely technically the sample variance of the correlation coefficients decreases when the true correlation increases [34]. Thus, the increase in the voxel-wise MAE values with the average ISC means that subject-pair-to-subject-pair variability of ISC generally increases with increasing average ISC. We note that this phenomenon was independent of the applied sample size and particularly all the MAE maps, uploaded to http://www.neurovault.org/collections/WTMVBEZP/, were virtually identical except for the scale of MAE values.

The data in this study was based on a traditional block design stimulus while the ISC analysis is typically applied for fMRI data with naturalistic stimuli. This choice was made out of necessity since no large enough naturalistic stimulation studies exist. In principle, the block design data might have limitations not to reveal all sources of variation involved in the ISC analysis. In particular, the data involves the replication of the same task/stimulus pattern and therefore might lead to positively biased reliability measures for the naturalistic stimulation fMRI. On the other hand, we have shown that ISC is applicable to block design data [10, 22], which partially justifies the use of block design data. Also, it should be noted that the naturalistic stimuli themselves are highly varied and therefore using one type of naturalistic stimuli might have the same limitations as our use of the block design stimulus. Due to high computational demands of the analysis, we chose to only consider fMRI time series of certain length albeit the minimal length of the time series is an important consideration especially to the so-called time-window ISC analysis [2, 35]. To render the analysis more targeted towards the naturalistic stimulation studies, where one may stipulate that individual reactions to the used stimuli may differ more among the participants than with traditional fMRI setups, we included subjects with a wide age range spanning from 19 to 80 years to our analysis (see [36] for the age-effects on the verb generation task). We also included left-handed and ambidextrous subjects, which may be slightly controversial due to greater prevalence of right-lateralized language among the left-handed subjects (see [37] and references therein). However, most left-handers have left-lateralized language and there exist multiple other reasons not to exclude left-handers from neuroimaging studies [37].

The results of our split-half resampling analysis indicated that 20 subjects were the minimum number of subjects to achieve somehow reproducible ISC statistical maps, but for a good reproducibility it would be preferred to have 30 subjects or more. With 20 subjects, the correlation measure (*C*_avg_ (2)) was 0.82 (see Figure 2), the average MAE (*M*_avg_ (4)) was 0.015 (see Figure 3), and the average Dice coefficient was 0.71, 0.72, and 0.70 for *q* = 0.05, 0.01, and 0.001, respectively (see Figure 5). When the number of subjects was below 20, our analysis indicated weak reproducibility (see Figures 2, 5, and 3). The reproducibility improved clearly when the number of subjects was incremented from 20 to 30 (*C*_avg_ increased to 0.89, *M*_avg_ decreased to 0.010, and the average Dice coefficient increased to 0.74, 0.77, and 0.78, resp.), but adding more than 30 subjects did not improve the reproducibility so steeply any more. The average correlation between the subsample ISC statistical map and the whole sample ISC statistical map was 0.92 already with 20 subjects and 0.95 with 30 subjects indicating that ISC statistics maps converged rapidly towards the whole sample ISC maps. As seen in Figure 7, the average sensitivity of the ISC detection, when compared to the thresholded ISC map with 130 subjects, was not particularly high even with 30 subjects. However, the specificity of ISC detections was close to 1 indicating that nearly all voxels detected with small sample sizes were also detected in the full 130-subject sample. This is not surprising and largely replicates the findings for the GLM based analysis of the event related GO/NOGO task in [17]. Also, our results were in line with the studies on the reproducibility in the GLM based analysis [16] recommending that more than 20 or even more than 30 subjects should be used in fMRI group analysis. Obviously, how many subjects are required for a particular fMRI study ultimately depends on the experiment and the guidelines provided by this work may not be applicable for all experiments involving ISC analysis.

## 5. Conclusions

We studied the effect of sample size for ISC analysis to determine how many subjects are needed for a reliable ISC analysis. We also investigated how small sample is enough for the ISC statistic to converge to ISC statistic obtained with a large sample. We found that with 20 subjects the ISC statistics were converged close to a large 130 subjects' ISC statistic. However, the reliability of unthresholded and thresholded maps improved notably when the number of subjects was increased to 30 subjects, which indicated that with this data 30 subjects or more should be used with ISC analysis for truly reproducible results. Finally, we emphasize that the required number of subjects depends on the specific characteristic of the experiment, including the expected effect size.

## Additional Material

Three-dimensional statistical maps are available in the NeuroVault service: http://www.neurovault.org/collections/WTMVBEZP/.
